# Tübingen hip flexion splints for developmental dysplasia of the hip in infants aged 0–6 months

**DOI:** 10.1186/s12887-020-02171-0

**Published:** 2020-06-05

**Authors:** You Zhou, Rong Li, Chuan Li, Ping Zhou, Yan Li, You-hao Ke, Fei Jiang, Xiao-peng Kang

**Affiliations:** 1 Department of Orthopedics, Kunming Children’s Hospital, 288 Qianxing Road, Xishan District, Kunming, 650034 Yunnan China; 2 Department of Obstetrics, First Affiliated Hospital of Kunming Medical University, Kunming, 650000 China; 3 Department of Orthopedics, People’s Liberation Army Joint Logistic Support Force 920th Hospital, Kunming, 650032 China

**Keywords:** Developmental dysplasia of the hip, Tübingen splint, Ultrasonography, Graf classification

## Abstract

**Background:**

Developmental dysplasia of the hip (DDH) is a common disorder in infants. The present study aimed to evaluate the efficacy and safety of the Tübingen hip flexion splints in treating DDH in infants aged 0–6 months.

**Methods:**

This is a retrospective study analyzing 259 hips in 195 infants with DDH of Graf type IIc or worse classifications treated between January 2015 and December 2017. Patients were followed up for at least 6 months. Avascular necrosis of the femoral head was diagnosed using plain radiographs at the last follow-up visit according to the Bucholz-Ogden classification. Successful treatment was defined as an improvement of the Graft classification to type I, or an improvement of the International Hip Dysplasia Institute classification to type I in patients aged > 6 months.

**Results:**

Treatment was deemed successful in 128 patients (83.7%). Avascular necrosis occurred in 3 patients (3 hips). Univariate analysis showed that late treatment initiation, family history of DDH, Graf type IV and bilateral involvement were independent risk factors for treatment failure (*p* < 0.05). The receiver operating characteristic curve showed a cut-off value of 12 weeks for age at treatment initiation regarding successful treatment. Logistic regression analysis showed that gender, breech presentation, firstborn, swaddling, birth weight > 3.5 kg, oligohydramnios, foot deformity and torticollis did not affect the success rate of treatment (*p* > 0.05).

**Conclusions:**

The Tübingen splint showed good efficacy and safety in treating DDH in infants aged 0–6 months. Family history of DDH, Graf classification of type IV, bilateral involvement and treatment initiation after 12 weeks of age are risk factors of treatment failure.

**Trial registration:**

N/A

## Background

Developmental dysplasia of the hip (DDH) is a common disorder of hip deformity, with manifestations ranging from mild hip instability or acetabulum dysplasia to severe hip dislocation. Its reported incidence is 1.3–28.5 per 1000 infants per year [1, 2]. Delayed diagnosis and treatment of DDH may result in limping, hip pain, pelvic tilt, scoliosis and osteoarthritis. A proportion of 43% of end-stage osteoarthritis is associated with DDH [3]. In Norway, DDH patients accounted for 9% in first-time total hip arthroplasty, and 29% of the patients treated with this procedure under 60 years of age also had DDH [4]. Early diagnosis and treatment are critical to reduce risks of surgery and disability in patients with DDH. Ultrasonography examination is useful for the early diagnosis of DDH [5].

The Pavlik harness is the most commonly used method for the treatment of DDH in infants aged 0–6 months [6]. It allows a certain movement range of the hip and the knee while maintaining the hip joint in flexion and abduction, which is thought to promote the development of the femoral head and the acetabulum [7]. The reported success rate of reduction in DDH using the Pavlik harness is around 70% [8–10]. A study comparing the Pavlik harness, the Craig splint and the von Rosen splint found that the rigid von Rosen splint was associated with significantly less avascular necrosis and reoperation than the Pavlik harness [11]. The Tübingen splint is also a rigid splint and can maintain the flexion position of the hip while limiting its abduction [12]. In this position, the pressure in the hip is distributed evenly with less vessel tension. This may reduce the incidence of avascular necrosis associated with the treatment of DDH.

Our study aimed to evaluate the efficacy and safety of the Tübingen hip flexion splints in treating DDH in infants aged 0–6 months.

## Methods

This is a retrospective study analyzing the patients with DDH treated with the Tübingen hip flexion splints at our hospital between January 2015 and December 2017. Most of the patients were referred to our hospital from other cities. Only the Tübingen splint was used at our hospital. The inclusion criteria were as follows: Graf classification of type IIc, D, III and IV [5]; diagnosed with DDH before the age of 6 months; no other treatment except the Tübingen splint; has been followed up for at least 6 months. Patients with the following conditions were excluded: hip dislocation caused by neuromuscular diseases; suppurative arthritis of the hip associated with dislocation; complicated with other skeletal or muscular diseases.

The parents were educated on the use of the Tübingen splint and how to use it at home. The patients were placed in the Tübingen splints with hip flexion of 90–110° and hip abduction of < 60°. Patients with type IIc DDH wore the splints for at least 22 h daily with diaper changing and bathing if needed. Patients with type D, III or IV DDH wore the splints for 24 h daily and were evaluated every week. If the Graf classification was improved to type IIc or better, the splints were worn for at least 22 h daily with diaper changing and bathing if needed. Ultrasound examination was scheduled weekly for the first 3 weeks of treatment, then monthly until the 6th month of follow-up. Pelvic radiographs were taken at 6 months of age, the end of the treatment, 1 year of age, and 1.5–2 years of age. The splints were worn for an additional 1 month after normal ultrasound results of the hip. Then the treatment was stopped if ultrasound or pelvic radiography examination was normal.

Hip ultrasonography was classified using the Graf method [5] (Table 1). Hip radiography was classified according to the method proposed by the International Hip Dysplasia Institute (IHDI) [13]. The presence of avascular necrosis of the femoral head was assessed according to the Bucholz-Ogden classification [14]. Successful treatment was defined as an improvement of the Graft classification to type I, or an improvement of the IHDI classification to type I in patients older than 6 months without the need of open or closed reduction at the last follow-up. The treatment was deemed failure if the hip was not reduced after 3–4 weeks of treatment and required open or closed reduction.

**Table 1:** The Graf classification system of developmental dysplasia of the hip, based on sonographic angles of the hip

Type	Description	Bony Roof	Bony Rim	Cartilage Roof	α-angle	β-angle
I	Mature hip	Good	Angular/blunt	Covers the femoral head	≥60	< 77
IIa	Physiological (< 3 month)	Deficient	Rounded	Covers the femoral head	50–59	> 55
IIb	Delay of ossification (> 3 month)	Deficient	Rounded	Covers the femoral head	50–59	< 55
IIc	Critical hip	Severely deficient	Rounded to flattened	Still covers the femoral head	43–49	< 77
D	Decentering hip	Severely deficient	Rounded to flattened	Displaced	43–49	> 77
III	Dislocated hip	Poor	Flattened	Pressed upward, perichondrium slopes cranially	< 43	> 77
IV	Dislocated hip	Poor	Flattened	Pressed downward, perichondrium is horizontal or dips caudally	< 43	

Continuous data were presented as means and standard deviations. Categorical data were presented as percentages or frequencies. Comparisons were made using the Student’s *t*-test or the chi-square test. Risk factors of treatment failure were analyzed using the multivariate logistic regression model. Cut-off value of the age regarding treatment success or failure was calculated using the receiver operating characteristic (ROC) curve. *P* < 0.05 was considered statistically significant.

## Results

### Patient general information

**Fig. 1:**
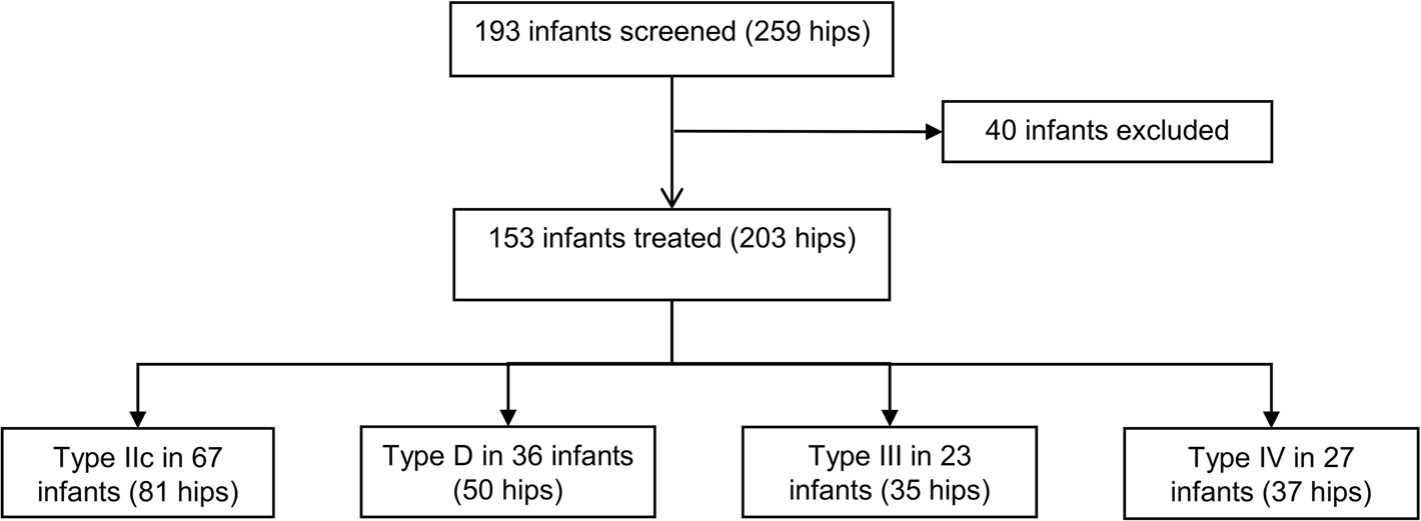
Diagram of patient inclusion

A total of 193 infants (259 hips) were initially screen and 40 patients were excluded due to comorbidities (7 patients), lack of baseline ultrasonography (5 patients), uncooperative parents (1 patient) and incomplete follow-up data (27 patients). Finally, 153 patients (203 hips) were included in our study with 26 boys and 127 girls (Fig. 1). The mean age at diagnosis and treatment initiation was 8.6 ± 5.6 weeks (range, 1–29 weeks). DDH affected only the left side in 76 patients, only the right side in 27 patients, and bilaterally in 50 patients. The Graf classification of the hips is shown in Table 2. The mean duration of treatment was 4.2 ± 2.2 months (range, 1–12 months). The mean follow-up time was 16.0 ± 7.7 months (range, 5–42 months). The treatment outcomes were considered successful in 128 patients (83.7%). Three patients (3 hips) with successful treatment were found to have mild avascular necrosis by radiography during follow-up 6 months after the treatment.

**Table 2:** Graf classification of the 203 hips and their treatment outcomes

Graf classification	Successful treatment (*n* = 165)	Failed treatment (*n* = 38)	*p*-value
IIc (%)	79 (47.9)	2 (5.3)	< 0.001
D (%)	46 (27.9)	4 (10.5)	0.035
III (%)	29 (17.6)	6 (15.8)	1
IV (%)	11 (6.7)	26 (68.4)	< 0.001

### Predictive factors and treatment outcomes

**Fig. 2:**
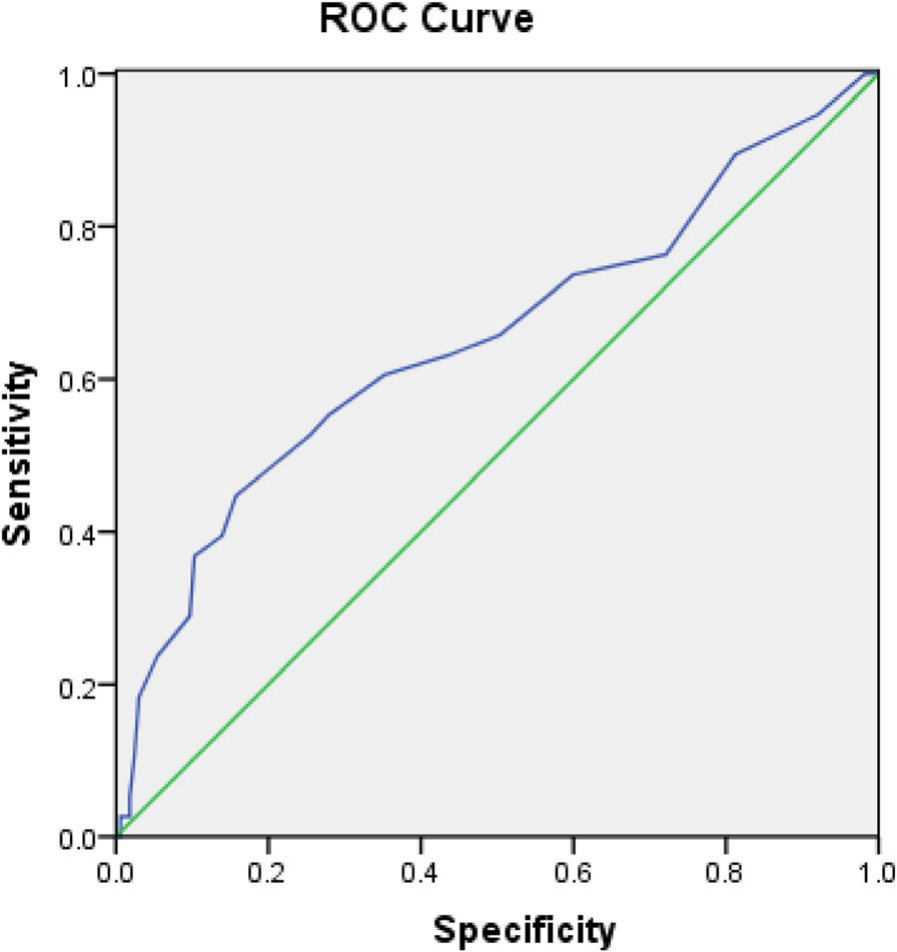
The receiver operating characteristic curve of age at treatment initiation regarding successful treatment

Comparison between infants with treatment success and those with treatment failures suggested that younger age at treatment initiation, unilateral hip involvement, no family history of DDH and Graf classifications < type IV might predict successful treatment with the Tübingen splints (Table 3). Multivariate logistic regression analysis suggested that age at treatment initiation, bilateral DDH and Graf classification were significantly associated with treatment outcomes (Table 4). Sex, breech presentation, firstborn, swaddling, birth weight > 3.5 kg, oligohydramnios, foot deformity and torticollis did not affect the success rate of treatment. The ROC curve showed that the cut-off value for age at treatment initiation regarding treatment success was 12 weeks, which yielded a sensitivity of 40% and a specificity of 88.3% (Fig. 2). The area under the ROC curve was 0.656 with a 95% confidence interval of 0.575–0.731.

**Table 3:** Comparison between DDH infants with treatment success and those with treatment failures

	Successful treatment (*n* = 128)	Failed treatment (*n* = 25)	*p*-value
Age at treatment initiation, week	8.0 ± 5.2	11.7 ± 6.6	0.020
Male, n (%)	23 (18)	3 (12)	0.663
Unilateral DDH, n (%)	91 (71.1)	12 (48)	0.024
Family history of DDH, n (%)	8 (6.3)	7 (28)	0.003
Breech infant, n (%)	27 (21.1)	8 (32)	0.235
Swaddling, n (%)	24 (18.7)	8 (32)	0.136
First born, n (%)	56 (43.8)	15 (60)	0.136
Oligohydramnios, n (%)	17 (13.3)	2 (8)	0.689
Birth weight > 3.5 kg, n (%)	13 (10.2)	0 (0)	0.203
Torticollis/foot deformity, n (%)	7 (5.5)	1 (4)	1.000
Graf type IV, n (%)	7 (5.5)	17 (68)	< 0.001

*DDH* developmental dysplasia of the hip

**Table 4:** Risk factors of failure in Tübingen splint treatment for DDH

	Correlation coefficient	S.E.	Wald	*p*-value	OR	95% CI
Age at treatment initiation	0.111	0.056	3.942	0.047	1.118	1.001–1.247
Gender	1.011	0.959	1.112	0.292	2.749	0.420–18.003
Right side DDH	0.839	0.896	0.877	0.349	2.315	0.399–13.417
Bilateral DDH	1.738	0.748	5.399	0.020	5.687	1.313–24.639
Family history of DDH	1.628	0.892	3.336	0.068	5.096	0.888–29.250
Graf classification	3.663	0.694	27.873	< 0.001	38.993	10.008–151.927

*DDH* developmental dysplasia of the hip, *OR* odds ratio, *CI* confidence interval

The treatment failed in 25 patients (38 hips). Two patients (2 hips) had Graf type IIc DDH. One of them was treated with a Tübingen splint for 2 months starting from the age of 4 months. The treatment was stopped when ultrasonography was normal. Radiography at 1-year follow-up showed an IHDI type II. The patient was then managed with plaster casting. Another patient had Graf type IIc DDH on the right side and Graf type III DDH on the left side. Ultrasonography of the right hip was normal after 3 weeks of treatment. However, the left hip showed no improvement and was managed with plaster casting. Two patients (4 hips) had Graf type D DDH, which worsened to type III after 1 week and 3 weeks of treatment, respectively. They were also managed with plaster casting. Six patients had Graf type III DDH on one side and Graf type IV DDH on the opposite side. Three hips were improved to Graf type II after 3 weeks of treatment. However, the opposite sides showed no improvement and were managed with plaster casting. One hip worsened to Graf type IV at 2-week and 3-week follow-ups. Twenty-six hips of the 37 hips with Graf type IV DDH failed the treatment.

### Case presentations

#### Case 1

**Fig. 3:**
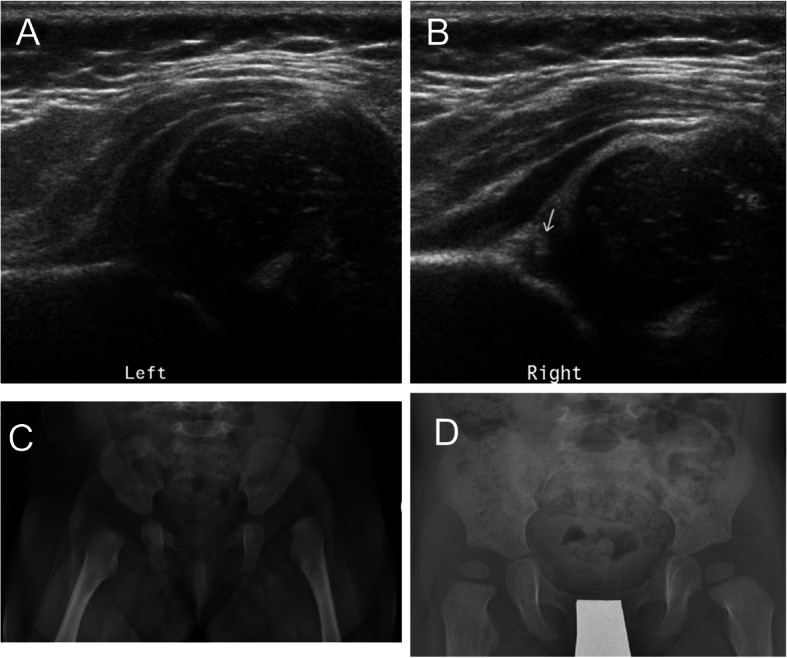
A 17-week-old girl who was the firstborn with breech presentation and swaddling

A 17-week-old girl was referred to our hospital who was the firstborn with breech presentation and swaddling. Shen had bilateral developmental dysplasia of the hip of Graf classification type IV on the left side (Fig. 3a) and type III on the right side (Fig. 3b) shown by ultrasonography. The patient wore a Tübingen splint for 1 month. X-ray showed International Hip Dysplasia Institute type III one the left side and type II on the right side (Fig. 3c). Then closed reduction with casting of the hip was performed. However, avascular necrosis of the left femoral head was fond by radiography 1 month later (Fig. 3d).

#### Case 2

**Fig. 4:**
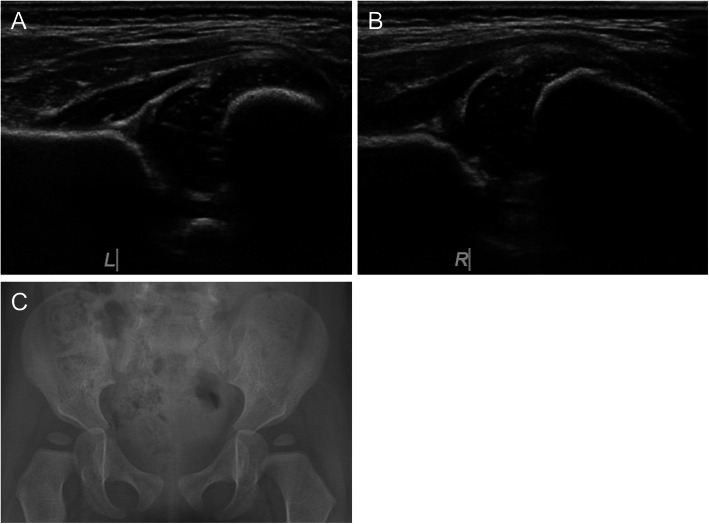
A 5-week-old girl with bilateral developmental dysplasia of the hips

A 5-week-old girl was referred to our hospital with bilateral developmental dysplasia of the hip of Graf classification type IIa on the left side (Fig. 4a) and type D on the right side (Fig. 4b) shown by ultrasonography. She wore a Tübingen splint for 2 months. Radiography showed normal development in both hips (Fig. 4c).

#### Case 3

**Fig. 5:**
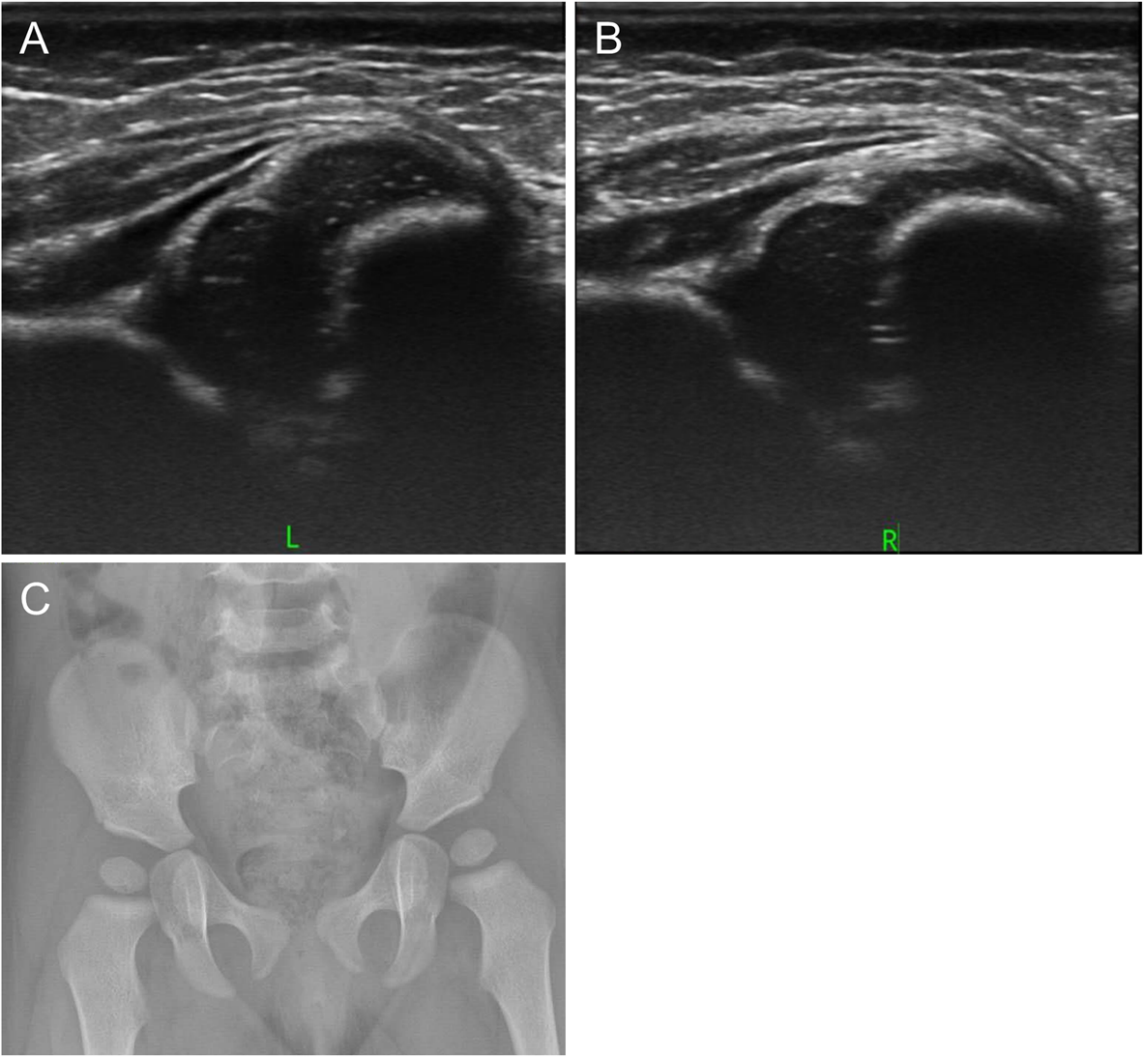
A 5-week-old girl with bilateral developmental dysplasia of the hips

A 5-week-old girl was referred to our hospital with bilateral developmental dysplasia of the hip of Graf classification type III on the left side (Fig. 5a) and type IIc on the right side (Fig. 5b) shown by ultrasonography. She wore a Tübingen splint for 3 months. Radiography showed normal development in both hips (Fig. 5c).

## Discussion

Possible risk factors of DDH include family history of DDH, breech presentation, oligohydramnios, birth weight > 3.5 kg, postterm pregnancy, firstborn baby and swaddling [14, 15]. In our study, 14/153 infants (9.2%) had family history of DDH and 6/14 of them (42.9%) failed the treatment. Our study showed that infants with family history of DDH had a significantly higher rate of treatment failure compared to those without family history. These findings support early screening of DDH if there is family history. In addition, many infants in our study had the following risk factors of DDH: breech presentation (23.5%), swaddling (20.9%), firstborn (47.1%), oligohydramnios (13.1%) and torticollis/foot deformity (5.9%). Our study did not find that these risk factors may affect the treatment outcomes of DDH using the Tübingen splint. However, infants with these risk factors should be screened for DDH.

In our study, the success rate of treating DDH of Graf type IIc and above before the age of 6 months using the Tübingen splint was 83.7%, which was comparable with previous studies (Table 5) [7, 16, 17, 19, 21]. Patient age at treatment initiation may influence treatment efficacy. Generally, older age at treatment initiation is associated with lower success rate of treatment. For example, Lin et al. [21] reported a 67.4% of success rate with patients starting the treatment at an age of 14.3 weeks, which is older than the age of 8.6 weeks in our study. In addition, our results showed that patients with successful treatment started using the splint significantly earlier by nearly 1 month than those with treatment failures. Older age of the infants promotes the maturity of the hip and reduce the bone plasticity required for successful splint treatment. Therefore, early screening in infants at increased risks of DDH and early treatment are critical for good outcomes. Unfortunately, DDH screening is still not a routine examination in China. Most of our patients were referred from other cities with probably delayed diagnosis.

**Table 5:** Summary of literatures for treating developmental dysplasia of the hip using the Tübingen splint

Year	Author	Patients (hips)	Age at treatment initiation (day)	Graf classification	Success	Failure	Success rate	Avascular necrosis
2012	T. Seidl [16]	42 (50)	3.5	IIc	6	0	49/50 (98%)	0
				D	33	0		
				III	10	0		
				IV	0	1		
2013	Bayalag Munkhuu [17]	99 (120)	1.9	IIc	35	1	95/97 (98%)	0
				D	70	0		
				III	14	1		
				IV	–	–		
2014	Hakan Atalar [18]	49 (60)	126	IIb	19	0	56/60 (93.3%)	0
				IIc	24	3		
				D	1	0		
				III	11	1		
				IV	1	0		
2014	Vito Pavone [19]	351 (554)	39	IIb	–	–	92.3%	3
				IIc	–	–		
				D	–	–		
				III	–	–		
				IV	–	–		
2017	Hannes Kubo [7]	79 (109)	< 42	D	51	0	104/109 (95.4%)	
				III	45	1		
				IV	8	4		
2018	Murat Yegen [20]	92 (104)	83	IIc	–	–	78 (75%)	0
				D	–	–		
				III	–	–		
				IV	–	–		
2019	Lin Ran [21]	34 (43)	100	IIb	18	0	29/43 (67.4%)	0
				IIc	7	3		
				D	0	0		
				III	1	0		
				IV	3	11		

Another affecting factor of treatment outcome of the Tübingen splint in DDH is disease severity. A previous multicenter prospective study showed a high treatment failure rate in patients with Graf type IV DDH [22]. The treatment failure rate for Graf IV DDH was 33.3% (4/12) and 78.6% (11/14) in two previous studies [7, 21]. In our study, the treatment failure rate in the Graf type IV hips was significantly higher than other Graf types (70.3% vs 7.2%, *p* < 0.001). The Tübingen splint is a rigid brace, which is different from the non-rigid Pavlik harness and is not capable of reducing severe hip dislocations. Reduction before wearing the Tübingen splint may be a possible technique to increase the success rate of hip reduction. However, this suggestion still needs clinical evidence.

Avascular necrosis of the femoral head is the most important complication in treating DDH. The reported incidence of avascular necrosis in DDH treated with the Pavlik harness was 0–30% [10, 23]. On the contrary, treating DDH with the Tübingen splint was found to be associated with relatively lower incidence of avascular necrosis [18–20]. In our study, 3 patients (2.0%) with 3 hips (1.5%) achieved successful treatment outcomes but were found to have mild avascular necrosis by follow-up radiography according to the Bucholz-Ogden classification. The relatively high incidence of avascular necrosis of the Pavlik harness may be attributed to the over abduction of the hip, which is associated with the non-rigid design of this orthosis. The rigid Tübingen splint can effectively limit the abduction of the hip and possibly reduce the incidence of avascular necrosis.

There are limitations in our study. Our study is a single-center retrospective study and lakes baseline physical examinations. Reducible and irreducible hip dislocations were indiscriminately included. The Tübingen splints were worn with varied time periods ranging from 20 to 24 h daily. Hip reduction was not required before treatment initiation. A high proportion of 17.7% of the patients were lost to follow-up. The short follow-up time may not reveal the true incidence of avascular necrosis. The ultrasonography was only viewed by a single specialist, which may give inaccurate diagnoses.

## Conclusions

The Tübingen splints showed good efficacy in treating DDH in infants aged 0–6 months with low risks of avascular necrosis of the femoral head. Family history of DDH, Graf classification of type IV, bilateral involvement and treatment initiation after 12 weeks of age may predict treatment failure. The Tübingen splint is a sensible option for treating DDH besides the Pavlik harness.

## Data Availability

All data generated or analyzed during this study are included in this published article.

## Abbreviations

DDH: Developmental dysplasia of the hip
IHDI: The International Hip Dysplasia Institute
ROC: The receiver operating characteristic

## References

[1] Morello P (2000). Clinical practice guideline: early detection of developmental dysplasia of the hip. Committee on quality improvement, subcommittee on developmental dysplasia of the hip. American Academy of Pediatrics. Pediatrics..

[2] Lee TW, Skelton RE, Skene C (2001). Routine neonatal examination: effectiveness of trainee paediatrician compared with advanced neonatal nurse practitioner. Arch Dis Child Fetal Neonatal Ed.

[3] Aronson J (1986). Osteoarthritis of the young adult hip: etiology and treatment. Instr Course Lect.

[4] Furnes O, Lie SA, Espehaug B, Vollset SE, Engesaeter LB, Havelin LI (2001). Hip disease and the prognosis of total hip replacements. A review of 53,698 primary total hip replacements reported to the Norwegian Arthroplasty register 1987-99. J Bone Joint Surg (Br).

[5] Graf R (1983). New possibilities for the diagnosis of congenital hip joint dislocation by ultrasonography. J Pediatr Orthop.

[6] Pavlik A (1957). Method of functional therapy with strap braces as a principle of conservative therapy of congenital dislocation of the hip in infants. Z Orthop Ihre Grenzgeb.

[7] Kubo H, Pilge H, Weimann-Stahlschmidt K, Stefanovska K, Westhoff B, Krauspe R (2018). Use of the Tubingen splint for the initial management of severely dysplastic and unstable hips in newborns with DDH: an alternative to Fettweis plaster and Pavlik harness. Arch Orthop Trauma Surg.

[8] Walton MJ, Isaacson Z, Mcmillan D, Hawkes R, Atherton WG (2010). The success of management with the Pavlik harness for developmental dysplasia of the hip using a United Kingdom screening programme and ultrasound-guided supervision. J Bone Joint Surg (Br).

[9] Grill F, Bensahel H, Canadell J, Dungl P, Matasovic T, Vizkelety T (1988). The Pavlik harness in the treatment of congenital dislocating hip: report on a multicenter study of the European Paediatric Orthopaedic society. J Pediatr Orthop.

[10] Saket T, Vivek G, Manoj R (2013). The Pavlik method: a systematic review of current concepts. J Pediatr Orthop B.

[11] Avci S (2002). The efficacy of the Pavlik harness, the Craig splint and the von Rosen splint in the management of neonatal dysplasia of the hip. J Bone Joint Surg (Br).

[12] Bernau A (1990). The Tubingen hip flexion splint in the treatment of hip dysplasia. Z Orthop Ihre Grenzgeb.

[13] Narayanan U, Mulpuri K, Sankar WN, Clarke NM, Hosalkar H, Price CT (2015). International hip dysplasia institute. Reliability of a new radiographic classification for developmental dysplasia of the hip. J Pediatr Orthop.

[14] Roposch A, Wedge JH, Riedl G (2012). Reliability of Bucholz and Ogden classification for osteonecrosis secondary to developmental dysplasia of the hip. Clin Orthop Relat Res.

[15] Kural B, Devecioğlu Karapınar E, Yılmazbaş P, Eren T, Gökçay G (2019). Risk factor assessment and a ten-year experience of DDH screening in a well-child population. Biomed Res Int.

[16] Seidl T, Lohmaier J, Hölker T, Funk J, Placzek R, Trouillier HH (2012). Reduction of unstable and dislocated hips applying the Tubingen hip flexion splint?. Orthopade..

[17] Munkhuu B, Essig S, Renchinnyam E, Schmid R, Wihelm C, Bohlius J (2013). Incidence and treatment of developmental hip dysplasia in Mongolia: a prospective cohort study. PLoS One.

[18] Atalar H, Gunay C, Komurcu M (2014). Functional treatment of developmental hip dysplasia with the Tubingen hip flexion splint. Hip Int.

[19] Pavone V, Testa G, Riccioli M, Evola FR, Avondo S, Sessa G (2015). Treatment of developmental dysplasia of hip with Tubingen hip flexion splint. J Pediatr Orthop.

[20] Yegen M, Atalar H, Gunay C, Yavuz OY, Uras I, Kaptan AY (2019). Reduction of the dislocated hips with the Tubingen hip flexion splint in infants. Int Orthop.

[21] Ran L, Chen H, Pan Y, Liu Q, Canavese F, Chen S. Comparison between the Pavlik harness and the Tübingen hip flexion splint for the early treatment of developmental dysplasia of the hip. J Pediatr Orthop B. 2019. 10.1097/BPB.0000000000000667. 10.1097/BPB.0000000000000667 31503108

[22] Upasani VV, Bomar JD, Matheney TH, Sankar WN, Mulpuri K, Price CT (2016). Evaluation of brace treatment for infant hip dislocation in a prospective cohort: defining the success rate and variables associated with failure. J Bone Joint Surg Am.

[23] Suzuki S, Kashiwagi N, Kasahara Y, Seto Y, Futami T (1996). Avascular necrosis and the Pavlik harness. The incidence of avascular necrosis in three types of congenital dislocation of the hip as classified by ultrasound. J Bone Joint Surg Br.

